# Virtual Biopsy for the Prediction of MGMT Promoter Methylation in Gliomas: A Comprehensive Review of Radiomics and Deep Learning Approaches Applied to MRI

**DOI:** 10.3390/diagnostics15030251

**Published:** 2025-01-22

**Authors:** Augusto Leone, Veronica Di Napoli, Nicola Pio Fochi, Giuseppe Di Perna, Uwe Spetzger, Elena Filimonova, Flavio Angileri, Francesco Carbone, Antonio Colamaria

**Affiliations:** 1 Department of Neurosurgery, Karlsruher Neurozentrum, Städtisches Klinikum Karlsruhe, 76133 Karlsruhe, Germany; augustoleone96@gmail.com (A.L.); uwe.spetzger@klinikum-karlsruhe.de (U.S.); francesco.carbone615@gmail.com (F.C.); 2 Faculty of Human Medicine, Charité Universitätsmedizin, 10117 Berlin, Germany; 3 Department of Neurosurgery, University of Turin, 10124 Turin, Italy; veronicadinapoli196@gmail.com (V.D.N.); fochinicola98@gmail.com (N.P.F.); 4 Division of Neurosurgery, “Policlinico Riuniti”, 71122 Foggia, Italy; colamariaa@gmail.com; 5 Department of Neuroradiology, Federal Neurosurgical Center, 630048 Novosibirsk, Russia; e.filimonova@alumni.nsu.ru; 6 Department of Neurosurgery, University of Messina, 98122 Messina, Italy; flavioangileri@gmail.com

**Keywords:** radiomics, deep learning, machine learning, O6-methylguanine-DNA methyltransferase, gliomas, non-invasive diagnostics

## Abstract

**Background/Objectives**: The methylation status of the O6-methylguanine-DNA methyltransferase (MGMT) promoter in gliomas has emerged as a critical biomarker for prognosis and treatment response. Conventional methods for assessing MGMT promoter methylation, such as methylation-specific PCR, are invasive and require tissue sampling. **Methods**: A comprehensive literature search was performed in compliance with the updated PRISMA 2020 guidelines within electronic databases MEDLINE/PubMed, Scopus, and IEEE Xplore. Search terms, including “MGMT”, “methylation”, “glioma”, “glioblastoma”, “machine learning”, “deep learning”, and “radiomics”, were adopted in various MeSH combinations. Original studies in the English, Italian, German, and French languages were considered for inclusion. **Results**: This review analyzed 34 studies conducted in the last six years, focusing on assessing MGMT methylation status using radiomics (RD), deep learning (DL), or combined approaches. These studies utilized radiological data from the public (e.g., BraTS, TCGA) and private institutional datasets. Sixteen studies focused exclusively on glioblastoma (GBM), while others included low- and high-grade gliomas. Twenty-seven studies reported diagnostic accuracy, with fourteen achieving values above 80%. The combined use of DL and RD generally resulted in higher accuracy, sensitivity, and specificity, although some studies reported lower minimum accuracy compared to studies using a single model. **Conclusions:** The integration of RD and DL offers a powerful, non-invasive tool for precisely recognizing MGMT promoter methylation status in gliomas, paving the way for enhanced personalized medicine in neuro-oncology. The heterogeneity of study populations, data sources, and methodologies reflected the complexity of the pipeline and machine learning algorithms, which may require general standardization to be implemented in clinical practice.

## 1. Introduction

High-grade gliomas (HGGs) represent the most aggressive and prevalent primary brain tumors, marked by a poor prognosis. Methylation of the O6-methylguanine-DNA methyltransferase (MGMT) gene promoter has emerged as a key prognostic biomarker and a predictor of treatment response to alkylating agents, including temozolomide (TMZ) [1,2,3]. Patients with a methylated MGMT promoter generally experience better outcomes and exhibit greater sensitivity to these chemotherapeutic agents [4,5,6]. Consequently, MGMT promoter methylation status has become integral to clinical decision-making in glioma management, informing treatment protocols and potentially improving survival rates [7,8].

Conventional assessment of MGMT promoter methylation relies on techniques such as methylation-specific polymerase chain reaction (MSP), pyrosequencing, and other molecular assays [9,10]. However, these methods require invasive tissue sampling, exposing patients to anesthesiologic and surgical risks. Moreover, intratumoral heterogeneity can undermine the reliability of a single tissue sample, as methylation status may vary across different tumor regions [11,12,13,14]. These limitations underscore the need for alternative, non-invasive methods that can accurately evaluate MGMT promoter methylation.

In recent years, machine learning (ML)—specifically radiomics (RD) and deep learning (DL)—has gained attention for its ability to predict molecular markers, including MGMT promoter methylation, from standard imaging data, such as magnetic resonance imaging (MRI) [13,14,15,16]. RD involves extracting high-dimensional quantitative features from medical images, revealing tumor characteristics not visible to the naked eye. By contrast, DL architectures can discern complex patterns, facilitating predictions of molecular features, such as methylation status, that would otherwise require invasive testing [17,18,19,20].

This review provides a comprehensive, critical examination of the existing literature on RD- and DL-based methods for predicting MGMT promoter methylation in cerebral gliomas. We focus on the methodologies employed, the strengths and limitations of various RD and DL models, and their reported accuracies within clinical contexts. We also discuss challenges related to the clinical application of these techniques, including issues related to reproducibility, standardization, and interpretability of ML-derived results. Finally, we discuss future directions in this field, highlighting developments that are essential to enhance the reliability and clinical utility of non-invasive MGMT promoter methylation assessment. By offering clinicians and researchers a detailed overview of current ML applications in neuro-oncology, this review underscores the transformative potential of “virtual tumor sampling” to reshape diagnostic and prognostic strategies.

## 2. Objectives and Organization of This Review

The following are the main contributions to the literature of the present review:

- Identification of Gaps: This study highlights key gaps in existing methodologies, particularly regarding standardization, reproducibility, and clinical validation;
- Proposed Framework: We propose actionable recommendations for addressing barriers to integrating ML-based approaches into clinical workflows, including the adoption of standardized preprocessing protocols and data-sharing technologies;
- Emerging Technologies: We discuss how federated learning and blockchain can address challenges in data availability and security, enhancing collaborative research in this domain;
- Practical Applications: This review explores practical scenarios, such as pre-surgical planning and therapy response prediction, where RD-DL models can be effectively applied.

The remainder of this review is organized as follows:

- Section 3 details the materials and methods, including search criteria, inclusion and exclusion parameters, and the datasets reviewed;
- Section 4 discusses the findings, including performance metrics, challenges, and potential solutions;
- Section 5 focuses on emerging technologies and actionable recommendations for overcoming barriers to clinical adoption;
- Section 6 concludes this paper, summarizing key findings and outlining directions for future research.

## 3. Materials and Methods

**Figure 1:**
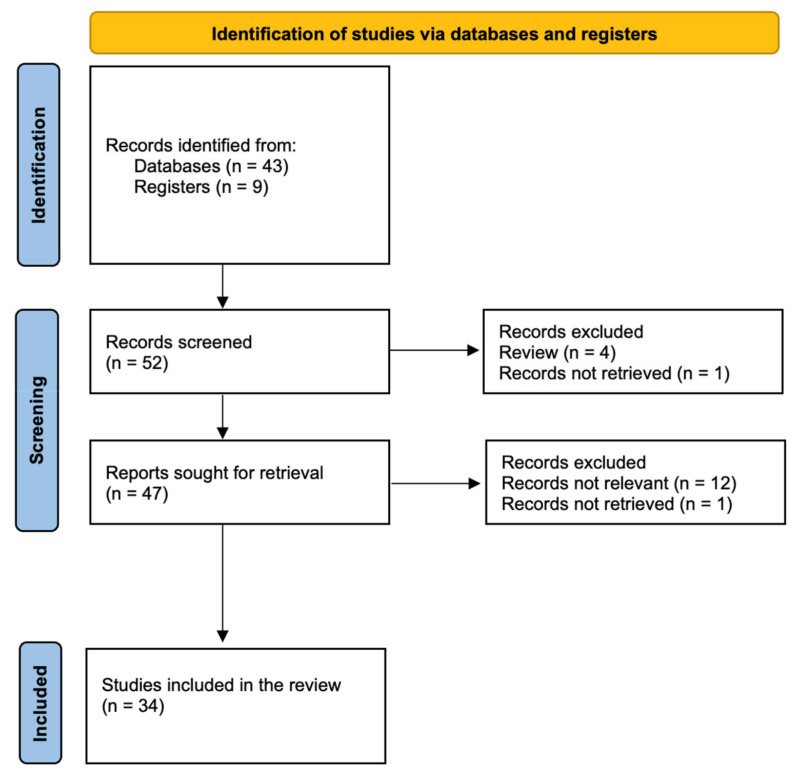
PRISMA 2020 flow diagram.

This review followed the PRISMA guidelines to identify and analyze studies employing RD or DL techniques to detect MGMT methylation status in cerebral gliomas. Figure 1 shows comprehensive searches conducted across multiple databases, including PubMed, Scopus, and IEEE Xplore, from January 2018 to September 2024. The choice of limiting this review to the articles published in the last six years was motivated by the advent of DL approaches for the grading and identification of molecular mutations using MRI data of gliomas [21]. The research was performed using various MeSH combinations of terms such as “MGMT”, “methylation”, “glioma”, “glioblastoma”, “machine learning”, “deep learning”, and “radiomics”. Studies were selected based on previously set inclusion criteria, including (1) investigation focusing on MGMT promoter methylation detection, (2) use of ML methods, (3) investigations based on T1 weighted images (T1WI), T2WI, FLAIR, and T1WI with gadolinium (-Gd), and (4) adequate data reporting for performance evaluation. Exclusion criteria included (1) studies not related to MGMT promoter methylations and (2) investigations based on ML applied to imaging modalities different than MRI. Data extracted from each study included ML model type, dataset size, feature selection methods, pre-/and processing pipeline, performance metrics (e.g., accuracy, sensitivity, specificity), and any validation techniques used. From this review, a total of 18 articles were excluded (four were reviews on the topic; two articles were not retrievable from the Journal website, and twelve articles were deemed out of this review scope as they examined other imaging techniques, including PET-CT, or focused on the grading of gliomas rather than the prediction of MGMT promoter methylation).

### 3.1. Datasets Utilized in Reviewed Studies

The reviewed studies predominantly utilized datasets from both public and private sources. Public datasets such as the Multimodal Brain Tumor Image Segmentation Benchmark (BraTS) and The Cancer Genome Atlas (TCGA) were frequently cited. BraTS provides high-quality, multi-institutional MRI scans with annotations for glioma segmentation, making it a valuable resource for training and evaluating radiomics and deep learning models. TCGA complements this with an extensive repository of molecular and imaging data, including information on MGMT promoter methylation, facilitating the development of radiogenomic approaches.

Private datasets, typically derived from institutional studies, varied in size and imaging protocols, often lacking the consistency and standardization found in public datasets. These datasets were valuable for exploring novel hypotheses but presented challenges related to reproducibility and generalizability.

Each dataset included in this review was selected based on its relevance to ML-based glioma research, its ability to support the study’s objectives, and its accessibility to the broader research community. By outlining the characteristics and limitations of these datasets, we aim to provide a resource that aids researchers in selecting appropriate data sources for future investigations.

### 3.2. Commonly Used Performance Metrics

In the context of ML applications for predicting MGMT promoter methylation, it is crucial to evaluate model performance using standardized metrics. The following are commonly employed metrics in the reviewed studies:

1. Accuracy: The proportion of correct predictions (both positive and negative) out of the total number of cases. It provides an overall measure of the model’s performance.

*Accuracy* = (*True Positives* + *True Negatives*)/*Total Cases*;

2. Sensitivity: The ability of the model to correctly identify positive cases (e.g., methylated MGMT promoter). It reflects the model’s capacity to minimize false negatives.

*Sensitivity* = *True Positives*/(*True Positives* + *False Negatives*);

3. Specificity: The ability to correctly identify negative cases (e.g., unmethylated MGMT promoter), highlighting the model’s precision in avoiding false positives.

*Specificity* = *True Negatives*/(*True Negatives* + *False Positives*);

4. Precision (Positive Predictive Value): The proportion of true positive predictions out of all positive predictions made by the model, measuring its reliability in identifying positives.

*Precision* = *True Positives*/(*True Positives* + *False Positives*);

5. Area Under the Receiver Operating Characteristic Curve (AUC-ROC): A metric evaluating the model’s ability to discriminate between classes across different thresholds. Higher AUC values indicate better model performance.

Each of these metrics provides unique insights into model performance, ensuring a comprehensive assessment of its predictive capability. Employing a combination of these metrics facilitates the comparison of methods and aids in identifying the most clinically applicable models.

## 4. Results

Thirty-four studies conducted between January 2018 and September 2024 were analyzed. Both private and public datasets provided data for these studies, and different RD and/or DL algorithms were investigated to assess sensitivity, specificity, and accuracy in the prediction of MGMT promoter methylation status. The characteristics of these studies are summarized in Table 1. An initial evaluation reveals significant heterogeneity among the study populations: some studies focused exclusively on HGG, while fourteen investigations included low (LGG) and HGG.

In fourteen studies, data were directly obtained from public repositories such as TCIA [22], TCGA [23], and the BraTS [24]. Although relying on similar radiological data, different software, preprocessing pipelines, and classifiers were applied, yielding diverse values of sensitivity, specificity, and diagnostic accuracy. Sixteen studies used data from private hospital datasets, and three studies combined imaging data from public sources with private hospital datasets, achieving notable diagnostic accuracy [16,25,26].

**Table 1:** Summary of tumor and MRI sequences characteristics.

Author	Year	Type of Dataset	Glioma Grades	Used Sequences
Han et al. [27]	2018	Public: TCIA and TCGA	HGG	T1WI, T2W2; T2 FLAIR
Li et al. [16]	2018	Public and Private TCIA and three local institutions	HGG	T1W1; T1Gd; T2-FLAIR
Xi et al. [28]	2018	Private	HGG	T1W1; T1Gd; T2WI
Hajianfar et al. [15]	2019	Public: TCIA	HGG	T1Gd; T2 FLAIR
Jiang et al. [29]	2019	Public—The Cancer Genome Atlas Low-Grade Glioma	LGG	T1Gd; T2WI
Sasaki et al. [30]	2019	Private	LGG + HGG	T1WI; T1Gd; T2WI; T2Edge; Gdzscore
Wei et al. [31]	2019	Private	LGG + HGG	T1Gd; T2-FLAIR; ADC
Calabrese et al. [32]	2020	Private	HGG	T1WI; T1Gd; T2-FLAIR; SWI; DWI; ASL; HARDI
Chen et al. [17]	2020	Public: TCIA and TGCA	HGG	T1WI; T1Gd; T2-FLAIR;
Le et al. [33]	2020	Public: TCGA and TCIA	HGG	T1WI; T1Gd; T2-FLAIR;
Lin et al. [34]	2020	Public: TCIA	LGG + HGG	T1WI; T1Gd; T2-FLAIR;
Lu et al. [35]	2020	Private	HGG	T1Gd
Haubold et al. [36]	2021	Private	LGG + HGG	T1WI; T1Gd; T2-FLAIR;
Huang et al. [37]	2021	Private	LGG + HGG	T1WI; T1Gd; T2WI; T2-FLAIR
Kihira et al. [38]	2021	Private	LGG + HGG	T1Gd; T2-FLAIR; DWI
Pasquini et al. [39]	2021	Private	HGG	MPRAGE; T2-FLAIR; DWI; ADC; PWI;
Sohn et al. [40]	2021	Private	HGG	T1WI; T1Gd; T2WI; FLAIR
Yogananda et al. [41]	2021	Public: TCAI and TCGA	LGG + HGG	T2WI
Zhang et al. [42]	2021	Private	LGG + HGG	T1WI; T1Gd; T2WI; T2-FLAIR
Do et al. [43]	2022	Public: TCIA and TGCA	HGG	T1WI; T1Gd; T2WI; T2-FLAIR
He et al. [44]	2022	Private	LGG + HGG	T1WI; T1Gd; T2WI; DWI; ADC
Kim et al. [45]	2022	Public: SNUH and BraTS 2021	LGG + HGG	T1WI; T1Gd; T2WI; T2-FLAIR
Pease et al. [46]	2022	Public: MDACC and TCGA	HGG	T1Gd; T2WI; T2-FLAIR
Doniselli et al. [47]	2023	Private	HGG	T1Gd—FLAIR
Faghani et al. [48]	2023	Public—BraTS2021	HGG	T1WI; T1Gd; T2WI; T2-FLAIR
Qureshi et al. [49]	2023	Public—BraTS 2021	LGG + HGG	T1WI; T1Gd; T2WI; T2-FLAIR
Saeed et al. [50]	2023	Public—BraTS 2021	LGG + HGG	T1WI; T1Gd; T2WI; T2-FLAIR
Saxena et al. [51]	2023	Public—BraTS 2021	LGG + HGG	T1WI; T1Gd; T2WI; T2-FLAIR
Sha et al. [26]	2023	Public: TCGA and TCIA + Private: FHSXMU and SPPH	LGG + HGG	T1Gd; T2-FLAIR
Zhong et al. [52]	2023	Three institutions	HGG	T1WI; T1Gd; T2WI
Guo et al. [53]	2024	Institutional	HGG	T1WI; T1Gd; T2WI; T2-FLAIR
Li et al. [25]	2024	Private + Public (TCAI)	HGG	T1WI; T1Gd; T2-FLAIR
Schimtz et al. [54]	2024	Public—TCIA	HGG	T1WI; T1Gd; T2WI; T2-FLAIR
Zheng et al. [55]	2024	Private	HGG	T1WI; T1Gd; T2WI; FLAIR; DWI; ADC

TCIA: The Cancer Imaging Archive, TCGA: The Cancer Genome Atlas; SNUH: Seoul National University Hospital; MDACC: MD Anderson Cancer Center; HGG: High-Grade Glioma; LGG: Low-Grade Glioma.

As shown in Table 1, a variety of MRI sequences—including T1WI, T1WI-Gd, T2WI, and FLAIR—have been employed across various studies. In some cases, diffusion-weighted imaging (DWI), including apparent diffusion coefficient (ADC) mapping, was also used. Additionally, Sasaki et al. [30] developed two image-processing techniques, Gdzscore and T2Edge, to improve the detection of MGMT promoter methylation in GBM. Specifically, Gdzscore enhances tumor visibility by calculating voxel-wise contrast between T1WI and T1WI-Gd sequences, while T2Edge applies a Prewitt filter to T2WI to accentuate edges, thereby improving tumor boundary delineation and facilitating accurate segmentation. In this study, both Gdzscore and T2Edge images were part of the preprocessing steps in the RD analysis pipeline, aimed at improving the quality and interpretability of the MRI data used for predictive modeling in GBM patients. Nevertheless, the authors were able to reach a peak accuracy of only 67%.

### 4.1. Preprocessing Pipeline

The preprocessing steps of image selection and normalization are a common and fundamental element in all studies, as illustrated in Table 2. This phase typically involves image segmentation, skull stripping, normalization, coregistration of images, RD, and/or DL. Each step in the pipeline performs a distinct operation, and the output of one step becomes the input for the next. A pipeline may present some useful features such as (1) sequential process, which organizes tasks in a specific order where the data flow from one stage to the next; (2) automation: once set up, the pipeline automates the process, reducing manual work; (3) modularity: each stage is a separate module or operation, making it easier to manage and adjust; (4) scalability: it can handle large amounts of data efficiently. On the other hand, pitfalls of organizing tasks in a pipeline include (1) complex setup, (2) limited flexibility, and (3) error propagation. Pipelines are highly effective for automating and scaling complex processes but can add complexity in setup and management. They are most useful when dealing with repetitive, large-scale tasks where consistency and efficiency are critical.

### 4.2. Skull Stripping

Skull stripping involves the removal of all non-brain tissues from MRI images. This process often requires specialized algorithms that identify and remove non-brain tissues, including Brain Extraction Tool (BET), Statistical Parametric Mapping (SPM), and Cancer Imaging Phenomics Toolkit (CaPTk). As shown in Table 2, the most commonly used tool in the studies was BET [32,34,36,41,42,46], while in twelve studies, the images were not subjected to this process, and in seven studies, it was not specified [16,25,29,38,44,48,54]. When applied correctly, skull stripping plays a crucial role, along with other preprocessing steps, in minimizing noise and bias.

### 4.3. Coregistration

Coregistration is an imaging technique to precisely align two or more images, ensuring they match up in terms of position, size, and orientation. It enables precise comparison and analysis across various types of scans and allows for accurate comparison and analysis across different types of scans or over different periods. By aligning images of the same area taken at different times, it becomes possible to track subtle changes, such as tumor growth or disease progression, with greater accuracy. This process is vital for combining data from various sources or longitudinal studies where images from the same subject are compared over time. In thirteen studies [15,17,27,31,32,33,35,37,43,46,51,52,53], the images were not co-registered, while in four studies, this was not specified. Software such as FSL (in seven studies), CaPTk, and ANTs were commonly used.

### 4.4. Normalization

Image normalization involves adjusting the data to improve comparability across different scans. In practice, normalization is typically performed during the preprocessing stage before the image is input into a model.

There are two main types of image normalization:

- Intensity normalization. This process adjusts the intensity values of MRI images to account for variations in pulse sequence parameters, magnetic field inhomogeneity, patient positioning, or other factors that can affect image brightness. The purpose is to make intensity values comparable across different scans, which is especially important in multicenter studies where different scanners are used. It usually involves two main steps: (1) convert the DICOM data to another format, with NifTI being the most popular option; (2) choose a normalization technique (N4 bias correction, Batch normalization, Z-score, etc.) to standardize the pixel values. By normalizing the data, inconsistencies in brightness or contrast can be removed, making it easier for the model to focus on the actual patterns in the image, improving the accuracy and performance of the analysis;
- Spatial Normalization. This method involves aligning the images to a standard template or coordinate system, using different registration techniques. The most widely used coordinate systems here are the Montreal Neurological Institute space template (MNI template) and the Talairach space template (Talairach atlas). Spatial normalization is crucial for comparison across subjects or for performing group analyses where scans need to be aligned in a standardized way.

Both types of normalization are essential for making MRI data reliable for quantitative analysis and machine learning applications. Among the examined studies, the normalization process is one of the most commonly applied, with only seven publications not providing specific information regarding this process [31,33,36,42,48,50,53]. The Z-score intensity normalization method was applied in eight studies, making it the most used method. Z-score, also known as a standard score, is a statistical measure that describes how far a data point is from the mean of a dataset, measured in terms of standard deviations. First, the mean (μ) and standard deviation (σ) of pixel values in the image are calculated. For grayscale images, the average pixel intensities are considered. Then, the Z-score formula is applied to transform each pixel (x) into a normalized value through a simple formula (x − μ)/σ; after this transformation, the image will have a mean of 0 and a standard deviation of 1, which helps in standardizing the data for analysis. This normalization can make models more efficient, especially when the images are used as inputs to ML algorithms, as it helps stabilize and speed up the learning process.

### 4.5. Segmentation

Image segmentation can be performed manually, semi-automatically, or automatically. In six studies [15,31,37,38,39,44], expert neuroradiologists performed manual segmentation, while in ten studies [26,28,29,34,35,41,46,55,56], manual feature selection by experts was paired with verification software. Certainly, the use of software inevitably minimizes interrater bias in tumor segmentation. In the BraTS challenge, various segmentation techniques are used, including traditional ML and advanced DL approaches. Recent experiences have demonstrated the effectiveness of various DL methods for tasks related to brain tumor segmentation and analysis [57,58,59,60], including (1) cascaded anisotropic convolutional neural networks (CNN), which have been used to improve segmentation accuracy by leveraging multiple stages of processing; (2) ML algorithms, which refer to a broader evaluation of these algorithms for brain tumor segmentation, progression assessment, and overall survival prediction, as seen in the BraTS challenge; (3) DL-based RD that involves extracting quantitative features from medical images to predict genetic biomarkers and analyzing them through ad hoc DL algorithms.

**Figure 2:**
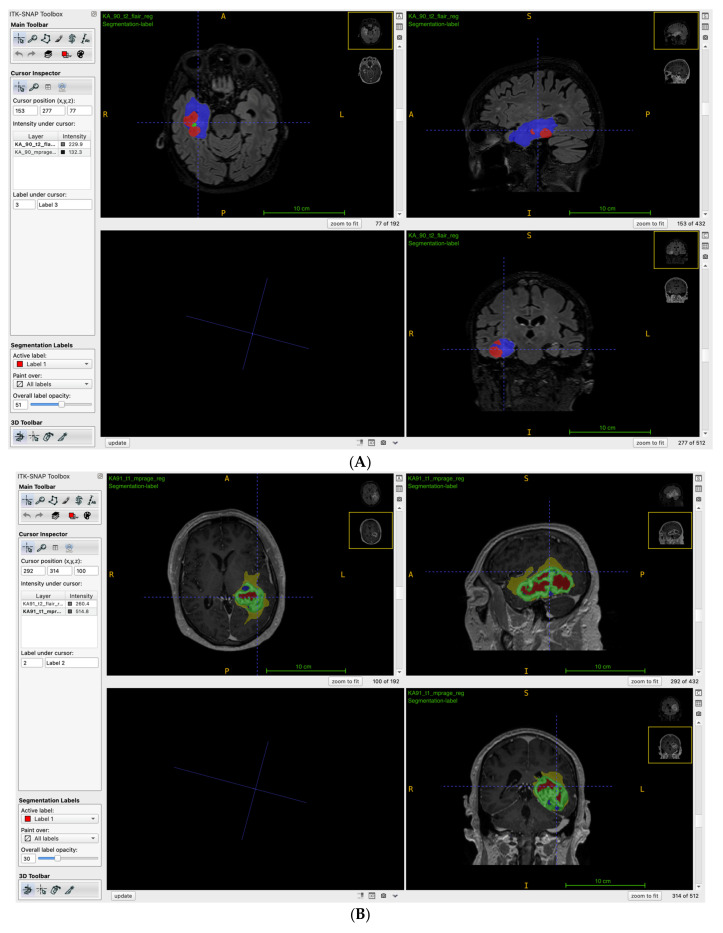
(**A**) ITK-SNAP Toolkit adopted for the semi-automatic segmentation of a right temporomesial and (**B**) large left temporal glioblastoma. Yellow: edema, green: contrast-enhancing component, red: necrosis.

Among the most common DL methods, CNN and U-Net are specifically designed to perform highly effective image segmentation. A similar method was used by Calabrese et al. [32], where a deep CNN (dCNN) for automated tumor segmentation was applied. Specifically, the segmentation network consisted of three cascaded instances of a 2-dimensional dCNN. The first instance was responsible for segmenting the entire tumor volume from whole brain images, while the second and third instances focused on segmenting the tumor core and the Gd-enhancing tumor, respectively, from the tumor volume. This approach allowed for rapid and accurate three-dimensional (3D) segmentation of GBM subregions from MRIs, which is crucial for subsequent RD feature extraction and genetic biomarker prediction. Notably, some authors applied morphological operations or Conditional Random Fields (CRFs) to refine the initial results to further improve the accuracy of the segmentation [24]. Furthermore, ITK-SNAP was utilized in six studies [25,26,29,54,55,56], and it is widely known for its capability in the semi-automatic segmentation of MRIs (Figure 2). The tool combines both manual input and automated algorithms, offering a balance between precision and efficiency in generating accurate segmentations. Its 3D visualization and region-growing algorithm allow for rapid segmentation with minimal manual effort, especially when defining regions of interest (ROIs). Unfortunately, as shown in Table 2, the segmentation process is not specified in five studies [27,32,48,53].

### 4.6. Radiomics vs. Deep Learning

As shown in Table 3, the software and classifiers used are also heterogeneous. Some studies used RD (*n* = 14; PyRadiomics, GLCM, HOG, LBP), while others adopted DL classifiers (*n* = 5; CNN, CRNN, UNet, ResNet), and some combined both approaches (*n* = 15). The most used tool for RD was PyRadiomics, an open-source Python package for extracting features from medical images to identify biomarkers, though three studies [33,34,50] did not specify the model used. Sha et al. [26] developed a model that successfully predicted a combination of the two factors: IDH mutation and MGMT promoter methylation in glioma. However, the focus of this study was not solely on predicting MGMT promoter methylation but rather on both genetic factors. Hence, the approach used in their study does not directly reflect the performance of MGMT methylation alone.

In seven studies [28,33,42,43,53,56], general ML was applied instead of DL; although the two techniques are similar in certain aspects, they are by no means identical. Indeed, ML involves algorithms that learn patterns from data and make decisions with minimal human intervention, while DL is a subset of ML that uses neural networks with many layers to automatically identify complex patterns in large datasets. Interestingly, better results were achieved using ML, and the most used algorithms are Extreme Gradient Boosting (XGBoost) in three studies [33,43,55] and the Support Vector Machine (SVM) model in two investigations [28,47]. The key difference is that XGBoost is an ensemble of learning methods that build decision trees iteratively, each one correcting errors of the previous ones in a process defined as “boosting”. SVM is a supervised learning algorithm that finds a hyperplane to separate data into classes. XGBoost is typically faster on large datasets due to its parallel capabilities, while SVM may be slower. Both algorithms are powerful, but XGBoost excels in structured data and large datasets, while SVM is better for specific classification problems with smaller datasets. However, comparing studies using DL and ML, the outcomes ultimately depend on the task and dataset. For instance, ML achieves better results for smaller, structured data and simpler tasks, while DL excels with large, unstructured data, like images, and complex tasks, like image recognition. DL requires more data and computing power but can outperform ML in those cases.

In the field of DL, CNNs are fundamental models for image-processing tasks. Over time, a variety of CNN architectures have been developed to tackle specific challenges and improve performance in different areas. Notable examples include LeNet for early image recognition, ResNet for addressing the vanishing gradient problem in deep networks, EfficientNet for optimizing model scaling, and UNet for precise image segmentation in medical and other applications. The best results were achieved by Qureshi et al. [49], where the authors highlight the application of the Deep Learning Radiomic Feature Extraction (DLRFE) module in predicting the genetic subtype of GBM, particularly the MGMT promoter methylation status, using multiparametric MRI. The DLRFE model extracts dynamic features, capturing spatial distribution and tumor size through a DL architecture [61]. It fuses these latent features with traditional RD features like Gray Level Co-occurrence Matrix, Histogram of Oriented Gradients, and Local Binary Patterns to create a hybrid feature set, enhancing the model’s predictive power [62]. This study reports improved classification performance using k-NN and SVM classifiers. Additionally, a novel rejection algorithm isolates negative training instances, refining the model’s accuracy [61,62]. In conclusion, the DLRFE module significantly contributes to better GBM classification and prediction outcomes. Unfortunately, the values for specificity, sensitivity, and accuracy were not determined for each study, making comparisons challenging. However, based on the data reported in Table 3, diagnostic accuracy was calculated in twenty-nine studies, with sixteen of them achieving values above 80%. In thirteen studies where both DL and RD approaches were employed, sensitivity and specificity were determined in only ten studies (sensitivity ranging from 64.29% to 96.18%, and specificity from 67% to 97.6%). In one study, diagnostic accuracy was not determined, while the remaining studies reported accuracy values varying from 45% to 96.8% [49]. When DL or ML was used without RD, diagnostic accuracy ranged between 56% and 94.7%, with specificity between 55.5% and 96.32% and sensitivity between 48% and 91.66% [41,48]. In fourteen studies that applied only RD, diagnostic accuracy varied between 65.3% and 89%, while sensitivity ranged from 46.9% to 90.5%, and specificity ranged from 65% to 93.6% [40,46]. Finally, the combined use of DL and RD yields higher maximum values for accuracy, sensitivity, and specificity compared to studies that used only one approach, even though the minimum accuracy values were lower in studies employing the combined approach.

## 5. Discussion

The methylation status of the MGMT promoter is a pivotal biomarker for predicting the efficacy of TMZ chemotherapy in GBM [63,64,65,66]. As conventional methods for assessing MGMT promoter status rely on invasive tissue biopsies, RD and DL offer promising non-invasive alternatives [41,67,68,69]. Leveraging imaging modalities such as multiparametric MRI, these methods aim to characterize tumor heterogeneity and predict molecular profiles accurately [70,71]. However, challenges such as variability in imaging protocols, feature extraction, and model generalizability need to be addressed for clinical adoption.

### 5.1. Radiomics: Current Developments and Challenges

RD has emerged as a powerful approach for extracting quantitative imaging features that correlate with tumor biology. Zheng et al. [55] demonstrated that RD models built using mpMRI—including T1WI, T2WI, and FLAIR—achieve significantly higher predictive accuracy (AUC 0.75) compared to single-sequence models. Similarly, Li et al. [25] developed a multiregional model incorporating features from enhancing, necrotic, and edematous regions, achieving AUCs of up to 0.84. This progress is complemented by Lin et al. [34], who highlight the potential of RD as a decision-support tool in the management of gliomas. The authors assert that RD, through quantitative analysis of medical imaging, can contribute to the risk stratification of glioma patients, enabling the prediction of survival outcomes. Specifically, their study demonstrated that an RD model derived from multiparametric MRI could distinguish subgroups of patients with HGG who exhibited significantly different prognoses. The analysis of RD features, such as those related to intensity, volume, morphology, histograms, and texture, facilitated the identification of two distinct patient subtypes with divergent prognostic outcomes. This ability to predict survival based on RD features could provide valuable insights for clinicians in personalized treatment planning, patient selection for specific therapies, and disease progression monitoring. The integration of RD data with clinical and molecular information could further enhance the accuracy of risk stratification and support more informed therapeutic decision-making [34].

### 5.2. Clinical Applications and Limitations

Despite these advancements, challenges persist in leveraging RD for MGMT promoter methylation prediction. Sasaki et al. [30] found that RD achieved an accuracy of only 67%, which was deemed insufficient for clinical utility. This is in contrast to the demonstrated success of RD in predicting IDH mutations in LGG and HGG [42,72,73,74]. This limitation may stem from the use of structural MRI alone, as evidenced by the study’s findings that second-order texture features, such as the standard deviation of GLRLM of the Gd-T1WI, while influenced by MGMT promoter methylation status, were inadequate for precise prediction [30,75,76]. Furthermore, while Sasaki et al. observed no asymmetry in lesion localization between methylated and non-methylated tumors, prior research highlighted the importance of lesion localization in predicting IDH mutations in LGG (grades I and II) [30,75,77,78].

Nevertheless, RD demonstrated clinical significance in stratifying GBM patients into prognostic groups independent of MGMT methylation status, with the RD risk score serving as an independent prognostic factor more robust than initial Karnofsky Performance Status or the type of surgery performed [30,79,80,81]. Most prognostic RD features were derived from the tumor core, a finding consistent with other studies. Additionally, combining the RD risk score with MGMT methylation status enhanced the stratification of clinical outcomes for newly diagnosed GBM patients, further underlining the integrative value of RD [30]. Further validating RD applications, a systematic review by Samartha et al. [82] and the work of Doniselli et al. [47] emphasized the clinical utility of RD in early, non-invasive MGMT promoter status estimation. Doniselli et al. [47] also demonstrated the advantages of integrating multiple tumor subregions, which improved sensitivity and specificity in MGMT methylation prediction. Standardization of imaging protocols is critical for advancing RD applications. Sasaki et al. [30] emphasized intensity normalization as essential for consistent quantitative analyses, aligning with clinical practices with radiologists adjusting image windows for improved interpretation [83,84]. Nyul et al. [85] proposed advanced methods for addressing scanner-related variability, enhancing downstream analyses such as segmentation and quantification. These steps are crucial for ensuring reproducibility and reliability in radiomic studies.

### 5.3. Deep Learning: Enhancing Radiomics

DL expands RD capabilities by enabling automated feature extraction and complex pattern recognition. Saxena et al. [51] introduced a fused DL paradigm combining handcrafted and deep features, achieving a 15% improvement in predictive performance over standalone RD models. Similarly, Sha et al. [26] demonstrated that integrating clinical variables with RD features in DL frameworks significantly enhanced MGMT promoter methylation prediction accuracy (AUC > 0.93). Li et al. [16] further explored DL and RD integration, emphasizing the utility of “all-relevant” models based on the Boruta algorithm for feature selection. This approach accounts for interactions among features and outperformed univariate models, achieving robust validation performance. By leveraging the “all-relevant” model, this study demonstrated higher AUCs even in validation cohorts, suggesting greater generalizability and reliability across datasets [16,86,87,88]. Additionally, Li et al. [16] underscored the advantages of RD over biopsy-based approaches, as RD imaging assesses the entire tumor, addressing the molecular heterogeneity of GBM [76,89,90,91,92]. The importance of comprehensive preprocessing pipelines, including bias field correction, skull stripping, isotropic voxel resampling, rigid registration, and intensity standardization, was also highlighted as critical for ensuring quality and consistency in extracted features [16,24,93,94,95,96,97,98].

Building on these advancements, Han et al. [27] and Palsson et al. [99] proposed integrating tumor segmentation with RD and shape features in DL pipelines, enhancing prediction accuracy by incorporating spatial and morphological characteristics. An example of an integrated RD and DL pipeline can be seen in Table 4.

Calabrese et al. [32] advanced these efforts with a dCNN for automatic segmentation of key GBM components, achieving high accuracy and scalability. Their approach allowed for rapid segmentation (under 25 s per MRI) and utilized PyRadiomics to extract standardized features, facilitating comparisons across studies [32]. Schmitz et al. [54] proposed adaptive fine-tuning methods to customize DL models for individual patient profiles, enhancing model generalizability. Saeed et al. [50] emphasized the necessity of external validation datasets to ensure model reliability, reinforcing the importance of robust and reproducible methods in RD-driven prediction frameworks.

### 5.4. Real-Life Application of RD-DL Models in Neurosurgery and Neuro-Oncology

RD-DL models have demonstrated their utility in enhancing the precision of surgical interventions. Advanced segmentation algorithms like U-Net and DeepMedic enable accurate delineation of GBM subregions, including the enhancing tumor core, necrotic core, and surrounding edema. These tools assist surgeons in defining tumor margins more effectively, potentially improving the extent of resection while preserving healthy brain tissue [32,36].

Additionally, RD-DL models can integrate functional MRI data to predict tumor proximity to critical functional areas, such as motor and language regions [100]. This capability allows surgeons to plan safer resections that minimize postoperative deficits [29,34]. Furthermore, longitudinal monitoring of chemotherapy response is another domain where RD-DL models excel. By analyzing changes in RD features from serial MRI scans, these models can provide insights into treatment effectiveness, tumor progression, or recurrence [45,47]. RD-DL approaches have also been employed in radiotherapy planning. By correlating RD features with molecular profiles, these models can predict radiation sensitivity in gliomas, facilitating personalized adjustments to radiation doses. This precision minimizes collateral damage to healthy tissues while ensuring effective tumor control [37,52].

Finally, combining RD and DL models allows for the stratification of glioma patients based on survival probabilities and disease progression risks. These models integrate RD-DL models and clinical data to identify high-risk patients and guide personalized treatment protocols. Additionally, models leveraging radiogenomic insights enhance the prediction of outcomes by associating imaging phenotypes with molecular markers such as IDH mutation and MGMT methylation [31,49].

### 5.5. Radiogenomics: Bridging Imaging and Molecular Data

Radiogenomics bridges imaging phenotypes and molecular profiles, offering a holistic view of tumor biology. Qureshi et al. [49] demonstrated the integration of spatial imaging features with molecular markers in a radiogenomic classification system, achieving sensitivity and specificity exceeding 96%. Similarly, Faghani et al. [48] validated voxel-wise, slice-wise, and whole-brain DL models for MGMT promoter methylation prediction, identifying whole-brain approaches as the most effective due to their ability to capture global tumor characteristics. Xi and Sasaki highlighted the significance of texture features as predictors of MGMT methylation status, emphasizing their value in reflecting intratumoral heterogeneity [28,30]. Xi identified that 25 out of 30 features from Gd-T1WI and 16 of 19 features from T2WI were texture-related, making them key biomarkers for stratification [28]. Sasaki similarly found that 14 of 22 prognostic features, identified through Supervised Principal Component Analysis, originated from texture analyses of central GBM lesions, further underlining the importance of these features in prognostication [30].

Integrating RD with genomic data enhances survival stratification and personalized treatment approaches. Wei et al. [31] and Lin et al. [34] demonstrated that combining RD with genomic information improves risk stratification, enabling the identification of distinct glioma subgroups with divergent prognostic outcomes. Han et al. [27] reinforced the utility of advanced MRI-derived features in capturing tumor heterogeneity associated with MGMT methylation, while Lin et al. [34] highlighted the potential of multiparametric MRI-derived radiomic models in predicting survival outcomes. In this study, the authors emphasized that RD, through its quantitative analysis of features such as intensity, volume, morphology, histograms, and texture, can distinguish subtypes of glioma patients with significantly different prognoses [34]. These findings support the integration of RD data with clinical and molecular information to enhance the accuracy of patient stratification, guide personalized treatment planning, and monitor disease progression, advancing its role as a decision-support tool in glioma management.

### 5.6. Key Challenges and Recommendations

Despite their promise, RD and DL face challenges in reproducibility, generalizability, and clinical interpretability. Doniselli et al. [56] highlighted the need for adherence to reporting guidelines like TRIPOD and RD quality scores to ensure reliability and reproducibility in RD studies. Saeed et al. [50] and others have noted dataset heterogeneity as a significant barrier, increasing the risk of overfitting in DL models, particularly when applied to small, single-center datasets. Collaborative initiatives utilizing large-scale, publicly available datasets, such as BraTS, TCIA, and TCGA, enable more robust evaluations, improving model reliability across diverse populations [22,101,102].

Standardization of imaging protocols and feature extraction methods is imperative to address variability in MRI acquisition techniques and post-processing pipelines, which often leads to inconsistent results across studies. Validation on large, multicenter cohorts, as emphasized by Qureshi et al. [49] and others, is essential for establishing the generalizability of predictive models. Emerging technologies like federated learning and blockchain-based data-sharing systems offer secure solutions for collaborative research, maintaining patient privacy while enabling broader training datasets. Interpretability remains a critical barrier to the clinical adoption of DL-based methods. Tools like Grad-CAM and feature visualization require further refinement to elucidate decision-making processes effectively, as noted by Saeed et al. [50]. Transparency and explainability are essential for building clinician trust and facilitating regulatory approval for clinical use. Feature selection is another critical aspect, as emphasized by Jiang et al. [29,103,104]. Techniques like LASSO ensure that models focus on the most relevant features, avoiding overfitting and enhancing predictive power. Interestingly, Jiang et al. [29] observed that while patient age correlates with MGMT methylation, it was not among the top predictive features selected by LASSO, suggesting that MRI-derived radiomic features are more informative for prediction. This finding highlights the importance of prioritizing RD features that capture intratumoral heterogeneity and molecular complexity [29,75,103,105]. Spearman correlation offers an additional perspective on model accuracy. Xi et al. [28] applied this measure to assess the relationship between predicted values from an RD-based model and actual MGMT promoter methylation status, achieving a strong positive correlation (0.7399) by integrating features from T1WI, T2WI, and Gd-T1WI. This underscores the potential of quantitative metrics in evaluating RD model performance. In conclusion, while RD and DL hold immense promise for non-invasive MGMT promoter methylation prediction, addressing dataset heterogeneity, standardization, and interpretability will be pivotal for their successful clinical translation. Emerging technologies and collaborative efforts will play crucial roles in overcoming these challenges, ensuring the broader applicability of these tools in precision medicine.

#### 5.6.1. Addressing Standardization, Reproducibility, and Clinical Validation Barriers in Radiomics and Deep Learning

To advance the use of RD and DL in predicting MGMT promoter methylation, several barriers related to standardization, reproducibility, and clinical validation must be addressed.

- Standardization

Standardization is critical for ensuring consistency across studies. Developing and adopting unified imaging acquisition protocols, such as those aligned with Quantitative Imaging Biomarkers Alliance (QIBA) standards, is essential. Preprocessing pipelines, including skull stripping, normalization, and segmentation, should follow common methodologies using established tools like HD-BET or PyRadiomics. Defining RD features according to the Image Biomarker Standardisation Initiative (IBSI) will further enhance consistency. Additionally, data should be stored in uniform formats, such as NIfTI for MRI scans, to facilitate cross-study comparison. Open-source software and standardized templates for preprocessing and feature extraction will make these practices more accessible;

- Reproducibility

Reproducibility can be improved through transparent reporting and data sharing. Adhering to standards like TRIPOD (Transparent Reporting of a multivariable prediction model for Individual Prognosis Or Diagnosis) ensures detailed documentation of methods and results. Public datasets, such as BraTS and TCIA, should be used for model training and validation, while federated learning frameworks can enable insights sharing without compromising data privacy. Independent benchmark teams should validate published models on external datasets. Hosting models and pipelines on version-controlled repositories (e.g., GitHub) will allow others to replicate and build on existing work;

- Clinical Validation

Multicenter studies are essential for assessing model performance across diverse populations and imaging settings. These trials should include patients with both low- and high-grade gliomas to enhance generalizability. Integration with clinical workflows is another priority; user-friendly interfaces can enable clinicians to incorporate DL predictions into routine diagnostics. Regulatory approvals require collaboration with agencies like the FDA and EMA to define evaluation criteria and conduct post-market surveillance to monitor real-world performance. Interpretability tools such as SHAP or Grad-CAM can provide insights into model decisions, fostering trust among clinicians. Validation on external datasets ensures robustness before deploying models in practice.

#### 5.6.2. Emerging Technologies for Data Sharing and Collaboration

Advancements in data-sharing technologies, such as federated learning (FL) and blockchain, hold great promise in addressing key challenges in non-invasive MGMT promoter methylation prediction. Federated learning enables collaborative model training across multiple institutions without the need to exchange raw data [106,107,108]. This privacy-preserving approach aligns with regulations like GDPR and HIPAA, ensuring data confidentiality while fostering multicenter collaborations [109]. By training models on diverse datasets across institutions, FL enhances generalizability and reduces biases from single-institution data, ultimately improving model robustness.

Blockchain technology complements FL by providing a secure, decentralized infrastructure for data integrity and traceability [110]. Using an immutable ledger, blockchain can securely record preprocessing steps, data transactions, and model updates, ensuring reproducibility and fostering trust among stakeholders. Furthermore, blockchain can facilitate regulatory compliance by creating transparent audit trails, which are critical for the clinical deployment of ML models.

Despite their potential, these technologies face challenges. For FL, ensuring consistency in data quality across sites is critical. Robust preprocessing standards and optimized algorithms like federated averaging can address these issues. Blockchain, while offering security, requires lightweight protocols tailored for healthcare applications to reduce computational overhead. Seamless integration with existing medical systems through APIs is also necessary for adoption [111].

Integrating FL and blockchain into the predictive modeling pipeline can enhance collaboration, protect patient data, and establish reproducibility. These technologies provide a roadmap for overcoming current barriers, paving the way for robust, secure, and clinically viable models in neuro-oncology.

## 6. Limitations

One significant limitation of the generalizability of the described results is the heterogeneity of study populations and data sources. This review includes studies utilizing both public datasets (e.g., BraTS, TCGA) and private institutional datasets. This variability introduces inconsistencies, particularly because public datasets are often well-curated and standardized, while private datasets may differ in imaging protocols, quality, and patient demographics.

The lack of standardization in imaging protocols, feature extraction methods, and ML pipelines is another critical concern. RD and DL models heavily rely on preprocessing steps such as skull stripping, normalization, and image segmentation. However, the reviewed studies employed varied preprocessing techniques, and several studies did not clearly define these methods. This inconsistency affects the reproducibility of the findings and highlights the need for unified guidelines for ML-based radiogenomic research.

Another drawback is the variability in methodologies and reported metrics. Although we critically combined results from RD, DL, and hybrid approaches, the diagnostic accuracy, sensitivity, and specificity metrics are inconsistently reported in the original studies. This inconsistency hinders the ability to benchmark different approaches and identify the most effective methods for clinical use. Additionally, limited external validation of the predictive models discussed raises concerns about their robustness and applicability across diverse clinical settings.

Interpretability remains a key challenge, particularly for DL models. Despite achieving high accuracy in some studies, these models are often viewed as “black boxes,” making it difficult for clinicians to understand the basis of their predictions. This lack of transparency could hinder the adoption of DL models in clinical workflows, where explainability is critical for trust and decision-making.

Furthermore, we highlighted the overfitting risk associated with ML models trained on small, single-center datasets. Overfitting reduces the models’ generalizability to new datasets, especially those collected from different institutions. Moreover, many studies focus exclusively on HGGs, leaving a gap in understanding the predictive power of these methods for LGGs, which are less represented in the literature.

Data quality concerns are another limitation. In several studies, preprocessing steps like skull stripping, normalization, and coregistration are inadequately reported, which can lead to inconsistencies in the features extracted for analysis. Moreover, the computational demand and complexity of DL models pose practical challenges, particularly in resource-limited settings.

Finally, the exclusion of experiences published before 2018 may pose limits to the conclusions of this review. For instance, early research, although potentially using less advanced techniques, could provide valuable insights into the evolution of methodologies, challenges addressed, and key milestones achieved.

## 7. Conclusions

RD and DL offer transformative potential for non-invasive prediction of MGMT promoter methylation in gliomas. By combining RD features and DL models, hybrid approaches consistently achieve higher diagnostic accuracy, sensitivity, and specificity compared to standalone methods, addressing gaps in tissue-based diagnostics. Despite advancements, challenges like standardization of preprocessing, feature extraction, and model validation persist. Variability in imaging protocols and dataset heterogeneity hinder reproducibility and clinical adoption. Standardized methodologies, multicenter collaborations, and technologies like federated learning and blockchain for secure data sharing are critical to overcoming these barriers and ensuring robust, generalizable models.

Beyond molecular profiling, RD-DL pipelines have applications in treatment planning, response monitoring, and prognostic stratification, enabling precision medicine in glioma care. Developing interpretable models with tools like SHAP and Grad-CAM can build clinician trust and foster broader acceptance. Future research should focus on external validation with diverse datasets and exploring radiogenomic applications to bridge imaging and molecular data. By addressing standardization and reproducibility challenges, radiomics and DL can revolutionize glioma diagnostics, providing precise, non-invasive tools for advancing neuro-oncology and personalized medicine.

## Figures and Tables

**Table 2:** Summary of the preprocessing pipelines adopted.

Author	Segmentation Software/Algorithm	Skull Stripping Software	Coregistration Software	Image Normalization Software/Algorithm
Han et al. [27]	Not specified	Not mentioned	Not mentioned	Batch Normalization
Li et al. [16]	TensorFlow (https://www.tensorflow.org) accessed on 15 November 2024	Not specified	Not specified	N4ITK
Xi et al. [28]	Manual segmentation + MITK (https://www.mitk.org/wiki/The_Medical_Imaging_Interaction_Toolkit) accessed on 15 November 2024	FSL (https://fsl.fmrib.ox.ac.uk/fsldownloads_registration/) accessed on 15 November 2024	FSL	Nyul intensity normalization
Hajianfar et al. [15]	Manual Segmentation	Manually	Not mentioned	Not specified
Jiang et al. [29]	Manual segmentation + ITK-SNAP (http://www.itksnap.org/pmwiki/pmwiki.php) accessed on 15 November 2024	Not specified	FSL	Pyradiomics (https://pyradiomics.readthedocs.io/en/latest/) accessed on 15 November 2024
Sasaki et al. [30]	MatLab (https://www.mathworks.com/products/matlab.html) accessed on 15 November 2024	MatLab	FSL	FSL
Wei et al. [31]	Manual segmentation	Not mentioned	Not mentioned	Not mentioned
Calabrese et al. [32]	Not specified	BET (https://mangoviewer.com/plugin_jbet.html) accessed on 15 November 2024	Not mentioned	ANTs
Chen et al. [17]	BraTS 2018 + VAE	Not mentioned	Not mentioned	Batch Normalization
Le et al. [33]	BraTS	Not mentioned	Not mentioned	Not mentioned
Lin et al. [34]	Manual segmentation + GLISTR (https://www.nitrc.org/projects/cbica_glistr/) accessed on 15 November 2024	BET + MASS method	Not specified	Z-score
Lu et al. [35]	Manual segmentation + ITK SNAP (for necrosis)	Not mentioned	Not mentioned	CaPTk (https://www.med.upenn.edu/cbica/captk/) accessed on 15 November 2024
Haubold et al. [36]	BraTS 2019 pretrained DeepMedic network (https://deepmedic.org) accessed on 15 November 2024	HD-BET algorithm	SimpleITK extension SimpleElastix (https://simpleelastix.github.io) accessed on 15 November 2024	Not mentioned
Huang et al. [37]	Manual Segmentation	Not mentioned	Not mentioned	Z-score
Kihira et al. [38]	Manual segmentation	Not specified	Olea Sphere (https://www.olea-medical.com/en/) accessed on 15 November 2024	Olea Sphere
Pasquini et al. [39]	Manual segmentation	Manual segmentation—3D Slicer (https://www.slicer.org) accessed on 15 November 2024	FMRIB Linear Image Registration Tool from FSL (https://web.mit.edu/fsl_v5.0.10/fsl/doc/wiki/FLIRT.html) accessed on 15 November 2024	Python Standard Scaler package (https://www.python.org) accessed on 15 November 2024
Sohn et al. [40]	HD-GLIO	HD-GLIO	HD-GLIO	N4 bias correction + Z-score
Yogananda et al. [41]	Manual segmentation + 3D-IDH Network	BET	ANTs	Advanced Normalization Tools; N4 Bias Field Correction; Intensity Normalization
Zhang et al. [42]	NiftyNet platform (https://niftynet.io) accessed on 15 November 2024	BET	FSL	Not mentioned
Do et al. [43]	Not specified	Not mentioned	Not mentioned	Not specified
He et al. [44]	Manual Segmentation	Not specified	ITK-SNAP	Z-score
Kim et al. [45]	FSL	3D Slicer	3D Slicer	N4ITK
Pease et al. [46]	Manual segmentation + 3D Slicer	BET	Not mentioned	Nyul intensity normalization
Doniselli et al. [47]	Semi-automatic—ITK-SNAP	SPM12 (https://www.fil.ion.ucl.ac.uk/spm/software/spm12/) accessed on 15 November 2024	ANTs	Z-score
Faghani et al. [48]	Not specified	Not specified	Not specified	Not specified
Qureshi et al. [49]	CNN; U-Net; CRFs	CaPTk + FeTS tool	CaPTk + FeTS tool	L2-norm
Saeed et al. [50]	CNN; U-Net; CRFs	CaPTK	CaPTK	Not mentioned
Saxena et al. [51]	CNN; U-Net; CRFs	Not mentioned	Not mentioned	N4-bias correction method
Sha et al. [26]	Manual segmentation + ITK-SNAP	Not mentioned	FSL	Intensity Normalization + Z-Score
Zhong et al. [52]	BraTS	Not mentioned	Not mentioned	SimpleITK, Z score normalization
Guo et al. [53]	Not specified	Not mentioned	Not mentioned	Not mentioned
Li et al. [25]	3D U Net + ITK-SNAP	Not specified	MatLab	Z-score
Schimtz et al. [54]	BraTS	Not specified	Not specified	Min-max scaling
Zheng et al. [55]	Manual segmentation + ITK-SNAP	Not mentioned	FSL	PyRadiomics

MITK: Medical Image Toolkit; ITK-SNAP: Insight Segmentation and Registration Toolkit; BET: Brain Extraction Tool; FSL: FMRIB Software Library; ANTs: Advanced Normalization Tools; N4ITK: N4 Bias Field Correction; CaPTk: Cancer Imaging Phenomics Toolkit; FeTS: Federated Tumor Segmentation.

**Table 3:** Characteristics of radiomics and deep learning software used and their results.

Author	Radiomics Used	Deep Learning Used	Sensitivity	Specificity	Accuracy
Han et al. [27]	Not specified	CRNN	67%	67%	62%
Li et al. [16]	GLCM; GLRLM; GLSZM; NGTDM	-	-	-	80%(6f); 70%(8f)
Xi et al. [28]	Not specified	SVM	87.50%	75.00%	80%
Hajianfar et al. [15]	Shape—Intensity-Texture	-	-	-	-
Jiang et al. [29]	3D-CE-T1 Single Model; T2-weighted Single Model; Linear Combination Model; Fusion Radiomics Model; Clinical Integrated Model	-	71.4%; 82.1%; 92.9%; 82.1%; 92.9%	71.4%; 71.4%; 71.4%; 71.4%; 85.7%; 71.4%;	88.6%; 80%; 88.2%; 88.6%; 88.6%
Sasaki et al. [30]	Texture and Location analysis	-	-	-	67%
Wei et al. [31]	ROI segmentation, feature extraction, feature selection, and model construction	-	-	-	90.2%
Calabrese et al. [32]	PyRadiomics 2.2.0	-	-	-	-
Chen et al. [17]	-	CNN with VAE	-	-	82.70%
Le et al. [33]	Not specified	XGBoost	88%	88.7%	88.70%
Lin et al. [34]	Not specified	-	-	-	-
Lu et al. [35]	PyRadiomics	Not specified	-	-	45–67%
Haubold et al. [36]	PyRadiomics	HD-BET	75.6% ± 9.4%	81.5 ± 9.1%	78.6 ± 4.4%
Huang et al. [37]	Radscore	-	GBM: 90.5%, Gliomas: 70.2%	GBM: 72.7%, LGG + HGG: 90.6%	GBM: 78.3%, LGG + HGG: 83%
Kihira et al. [38]	First-order mean absolute deviation; GLCM	-	70%	65%	67%
Pasquini et al. [39]	PyRadiomics	-	-	-	70.8%
Sohn et al. [40]	PyRadiomics	-	46.9% BR; 47.7% ECC	77.7% (BR); 97.6% (ECC)	65.3% (BR); 76.1% (ECC)
Yogananda et al. [41]	-	3D-dense UNets	96.31%	91.66%	94.73%
Zhang et al. [42]	PyRadiomics 2.0.0	autoML with TPOT	81.1%	94%	89.40%
Do et al. [43]	-	XGBoost + GA; RF + GA; SVM + GA	89.4% (GBM); 78% (LGG)	96.6% (GMB); 62% (LGG)	92.5% (RF-GBM); 75% (LGG)
He et al. [44]	PyRadiomics	-	-	-	-
Kim et al. [45]	PyRadiomics	EfficientNet-B0 (CNN)	-	-	54.80%
Pease et al. [46]	Intensity-level histograms; GLCM; the Maximum Relevance Minimum Redundancy technique	-	84.60%	93.30%	89%
Doniselli et al. [47]	PyRadiomics 2.2.1	SVM—RF	83.5 ± 8.9%	82.5 ± 11.8%	83 ± 5.7% (SVM on CE-NEC-HYP)
Faghani et al. [48]	-	Not specified	(1) 71.2% (2) 55.5%; (3) 65.4%	(1) 58.9%; (2) 48%; (3) 51.5%	(1) 0.65; (2) 0.56; (3) 0.61
Qureshi et al. [49]	GLCM; HOG, LBP	DLRFE; HFS	96.08 ± 0.10%	97.44 ± 0.14%	96.84 ± 0.09%
Saeed et al. [50]	Not specified	ResNet, DenseNet, EfficientNet; ViT; Swin	-	-	-
Saxena et al. [51]	GLCM), GLRLM, LBP, NGTDM, GLSZM -> CaPTk	ResNet and EfficientNet	--		61.33% (ML); 69.26% (DL); 76.18% (Fused Deep Learning)
Sha et al. [26]	PyRadiomics	-	81.10%	80.80%	88.60%
Zhong et al. [52]	PyRadiomics	ResNet and C3D	64.29% (ResNet); 85.71% (C3D)	-	86.76% (ResNet); 89.71% (C3D)
Guo et al. [53]	-	PCA—FLD—Binary Hashing and Blockwise Histograms	-	-	70%
Li et al. [25]	uLR-mRMR-LASSO—ComRad	3D U Net	65%	95.70%	81.40%
Schimtz et al. [54]	Skewness; Energy; GLCM; GLSZM; GLSZM low gray; NGTDM	MedicalNet	78%	84%	81%
Zheng et al. [55]	PyRadiomics	XGBoost	-	-	75.4%

GLCM: Gray Level Co-occurrence Matrix; GLRLM: Gray Level Run Length Matrix; GLSZM: Gray Level Size Zone Matrix; NGTDM: Neighborhood Gray Tone Difference Matrix; CNN: Convolutional Neural Networks; SVM: Support Vector Machine; RF: Random Forest; XGBoost: eXtreme Gradient Boosting; DLRFE: Deep Learning Radiomic Feature Extraction; PCA: Principal Component Analysis; FLD: Fisher Linear Discriminant.

**Table 4:** Combined RD-DL Pipeline for MGMT Promoter Methylation Prediction.

Step	Description	Challenges and Solutions
1. Data Acquisition	Collect multiparametric MRI data (T1, T1-Gd, T2, FLAIR) from public and private datasets.	Challenges: Variability in imaging protocols. Solutions: Use intensity and spatial normalization.
2. Preprocessing	Prepare MRI data through skull stripping, segmentation, normalization, and bias field correction.	Challenges: Accurate segmentation and error propagation. Solutions: Use automated tools (e.g., HD-BET) and validate manually.
3. Radiomic Feature Extraction	Extract handcrafted features (e.g., texture, shape, intensity) using PyRadiomics or similar tools.	Challenges: Feature redundancy and segmentation errors. Solutions: Apply feature selection techniques like LASSO.
4. Deep Learning Feature Extraction	Train CNNs or use transfer learning to extract abstract features from MRI data.	Challenges: Large labeled datasets required. Solutions: Use data augmentation and federated learning.
5. Feature Fusion	Combine radiomic and deep learning features into a unified representation.	Challenges: Balancing scales and dimensions. Solutions: Normalize features and experiment with fusion strategies.
6. Model Training	Train hybrid models (e.g., Random Forest with fused features) and validate via cross-validation.	Challenges: Risk of overfitting. Solutions: Regularize models and use explainability tools like SHAP.
7. Validation and Testing	Test the model on external datasets to ensure generalizability.	Challenges: Dataset shifts and single-metric reliance. Solutions: Use comprehensive metrics and external validation.
8. Clinical Integration	Deploy the pipeline for non-invasive prediction in clinical workflows.	Challenges: Integration and interpretability. Solutions: Develop user-friendly interfaces and conduct pilot studies.

SHAP: SHapley Additive exPlanations.

## Data Availability

All data examined in this article are publicly available in the literature datasets.

## Abbreviations

ADC	Apparent Diffusion Coefficient
ANTs	Advanced Normalization Tools
AUC-ROC	Area Under the Receiver Operating Characteristic Curve
ASL	Arterial Spin Labeling
BET	Brain Extraction Tool
BraTS	Multimodal Brain Tumor Image Segmentation Benchmark
CNN	Convolutional Neural Networks
CRNN	Convolutional Recurrent Neural Network
DL	Deep Learning
FLAIR	Fluid-Attenuated Inversion Recovery
GBM	Glioblastoma
Gd	Gadolinium
HGG	High-Grade Glioma
ITK-SNAP	Insight Segmentation and Registration Toolkit
LGG	Low-Grade Glioma
MGMT	O6-methylguanine-DNA methyltransferase
ML	Machine Learning
MRI	Magnetic Resonance Imaging
NIfTI	Neuroimaging Informatics Technology Initiative
PCR	Polymerase Chain Reaction
PWI	Perfusion Weighted Imaging
PRISMA	Preferred Reporting Items for Systematic Reviews and Meta-Analyses
RF	Random Forest
RD	Radiomics
ROI	Region of Interest
SHAP	Shapley Additive Explanations
SVM	Support Vector Machine
SWI	Susceptibility Weighted Imaging
T1WI	T1 Weighted Images
T2WI	T2 Weighted Images
TCGA	The Cancer Genome Atlas
TRIPOD	Transparent Reporting of a Multivariable Prediction Model for Individual Prognosis or Diagnosis
VAE	Variational Autoencoder
XGBoost	Extreme Gradient Boosting
